# Socioeconomically Disadvantaged Neighborhoods Face Increased Persistence of SARS-CoV-2 Clusters

**DOI:** 10.3389/fpubh.2020.626090

**Published:** 2021-01-27

**Authors:** David De Ridder, José Sandoval, Nicolas Vuilleumier, Andrew S. Azman, Silvia Stringhini, Laurent Kaiser, Stéphane Joost, Idris Guessous

**Affiliations:** ^1^Geneva University Hospitals, Geneva, Switzerland; ^2^Faculty of Medicine, University of Geneva, Geneva, Switzerland; ^3^Laboratory of Geographic Information Systems (LASIG), School of Architecture, Civil and Environmental Engineering (ENAC), École Polytechnique Fédérale de Lausanne, Lausanne, Switzerland; ^4^Group of Geographic Information Research and Analysis in Population Health (GIRAPH), Geneva, Switzerland; ^5^Department of Epidemiology, Johns Hopkins Bloomberg School of Public Health, Baltimore, MD, United States

**Keywords:** SARS-CoV-2, COVID-19, socioeconomic inequalities, spatial clustering analysis, cluster persistence, transmission dynamics

## Abstract

**Objective:** To investigate the association between socioeconomic deprivation and the persistence of SARS-CoV-2 clusters.

**Methods:** We analyzed 3,355 SARS-CoV-2 positive test results in the state of Geneva (Switzerland) from February 26 to April 30, 2020. We used a spatiotemporal cluster detection algorithm to monitor SARS-CoV-2 transmission dynamics and defined spatial cluster persistence as the time in days from emergence to disappearance. Using spatial cluster persistence measured outcome and a deprivation index based on neighborhood-level census socioeconomic data, stratified survival functions were estimated using the Kaplan-Meier estimator. Population density adjusted Cox proportional hazards (PH) regression models were then used to examine the association between neighborhood socioeconomic deprivation and persistence of SARS-CoV-2 clusters.

**Results:** SARS-CoV-2 clusters persisted significantly longer in socioeconomically disadvantaged neighborhoods. In the Cox PH model, the standardized deprivation index was associated with an increased spatial cluster persistence (hazard ratio [HR], 1.43 [95% CI, 1.28–1.59]). The adjusted tercile-specific deprivation index HR was 1.82 [95% CI, 1.56–2.17].

**Conclusions:** The increased risk of infection of disadvantaged individuals may also be due to the persistence of community transmission. These findings further highlight the need for interventions mitigating inequalities in the risk of SARS-CoV-2 infection and thus, of serious illness and mortality.

## Introduction

There has been an active debate regarding the socioeconomic determinants contributing to the pandemic, several studies highlighting that the SARS-CoV-2 virus disproportionately affects socioeconomically disadvantaged individuals (1–3). Recent evidence has suggested that neighborhood environmental and socioeconomic factors including poor housing quality, overcrowding and inability to work from home may influence SARS-CoV-2 transmission (4). However, the association between neighborhood socioeconomic deprivation and SARS-CoV-2 transmission dynamics remains to be examined.

SARS-CoV-2 spreads via close contact during daily activities which results in geographic clustering of cases (5). The location and duration of persistence of these clusters—monitored using spatiotemporal cluster detection techniques—can provide unique insights into the determinants of transmission (6). We hypothesized that the increased risk of infection within disadvantaged communities is the result of conditions favoring sustained and persistent community transmission. Hence, socioeconomically deprived neighborhoods would have longer-lasting SARS-COV-2 transmission clusters than less deprived neighborhoods. We investigated this hypothesis by combining spatiotemporal cluster detection with cluster survival analysis, an approach similar to the one applied to cancer data by Huang et al. (7).

## Materials and Methods

We analyzed data from 3,355 SARS-CoV-2 RT-PCR positive test results among 17,698 individuals tested in the state of Geneva, Switzerland, covering the first phase of the pandemic (February 26 to April 30, 2020). All included patients were confirmed to be infected with SARS-CoV-2 by RT-PCR assays. The Virology Laboratory at Geneva University Hospitals did the tests and provided anonymized data, including residential addresses. Only individuals who provided a valid residential address and who resided state of Geneva were included.

Residential addresses were geocoded by address matching on the reference dataset of Swiss addresses (www.housing-stat.ch). SARS-CoV-2 transmission patterns were monitored through space and time using the modified space-time density-based spatial clustering of application with noise (6) (MST-DBSCAN) algorithm available in the Python package pysda. The MST-DBSCAN algorithm was run with a maximum spatial distance of 600 m, a minimum time-distance value of 1 day, and a maximum time-distance value of 14 days. We then replicated the analysis using different spatial windows (200, 400, 800, and 1,000 m) and observed similar results. The MST-DBSCAN algorithm is one among various density-based clustering methods to detect disease clusters. This modified version of the spatiotemporal DBSCAN presents the advantage of incorporating the effect of the incubation period. The MST-DBSCAN detects clusters of cases but also identifies the daily evolution type of each cluster (6). We projected the daily evolution type of space-time clusters onto the 2,830 Swiss Areas (SA) neighborhoods (www.microgis.ch) of the state of Geneva. This approach allowed to record the kind of cluster evolution each SA neighborhood underwent each day (i.e., increase, keep, decrease, no cluster). The SA neighborhood was used as it constitutes the smallest spatial unit characterized by aggregated census socioeconomic data in Switzerland.

An index of socioeconomic deprivation was calculated at the SA neighborhood-level using a method developed by Lalloué et al. (8) and used in various studies to analyze environmental and health inequalities. The socioeconomic data characterize the 2,830 SA neighborhoods of the state of Geneva and include data on occupation, education, median income, median rent, unemployment, and nationality. Principal component analysis was used to synthesize the information from these data. To obtain a single index for all neighborhoods, the inertia of the first component was maximized by discarding variables only weakly correlated with the first component and variables contributing lower than the average (8).

We defined spatial cluster persistence as the time in days from emergence to disappearance, and censored clusters remaining on the last day of the study period. Using spatial cluster persistence as the measured outcome, we estimated survival functions stratified by terciles of neighborhood-level socioeconomic deprivation with the Kaplan–Meier estimator. The contribution of the neighborhood-level socioeconomic deprivation to spatial cluster persistence was estimated in a Cox proportional hazards (PH) regression model (7) with robust standard errors. We then estimated the contribution of each individual component of the socioeconomic deprivation index [i.e., median income, foreigners (%), median rent, unemployment (%), occupation and education] as continuous independent variables in a separate Cox PH model (Table 1). Models were adjusted for neighborhood-level population density, and covariates were standardized.

**Table 1:** Hazard ratios and corresponding 95% confidence intervals for cluster persistence from a multi-variable Cox Proportional Hazards model.

**Covariate**	**HR [exp(b_**i**_)]**	**95% CI**
Median income	0.95	(0.93–0.97)
Foreigners (%)	1.23	(1.02–1.52)
Median rent	0.71	(0.61–0.83)
Unemployment (%)	1.14	(1.09–1.18)
Primary sector actives (%)	0.98	(0.96–1)
Tertiary education (%)	1.06	(0.87–1.3)

*HR, hazard ratio; CI, confidence interval*.

*Model adjusted for neighborhood-level population density to reduce potential confounding between covariates and spatial cluster persistence*.

## Results

**Figure 1:**
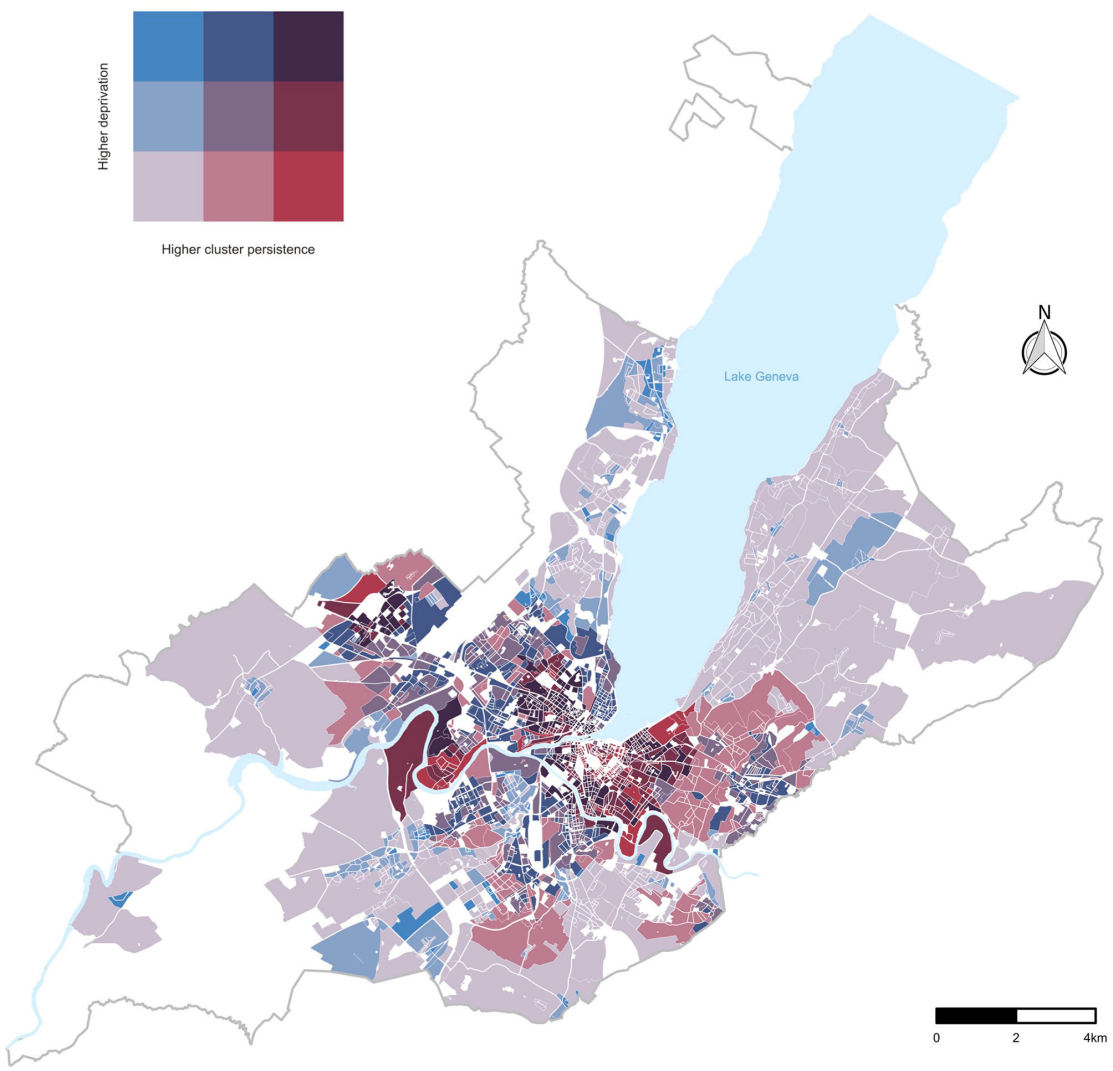
Bivariate choropleth map: Relationship between terciles of neighborhood-level deprivation index and of SARS-CoV-2 cluster persistence in the canton of Geneva, Switzerland.

**Figure 2:**
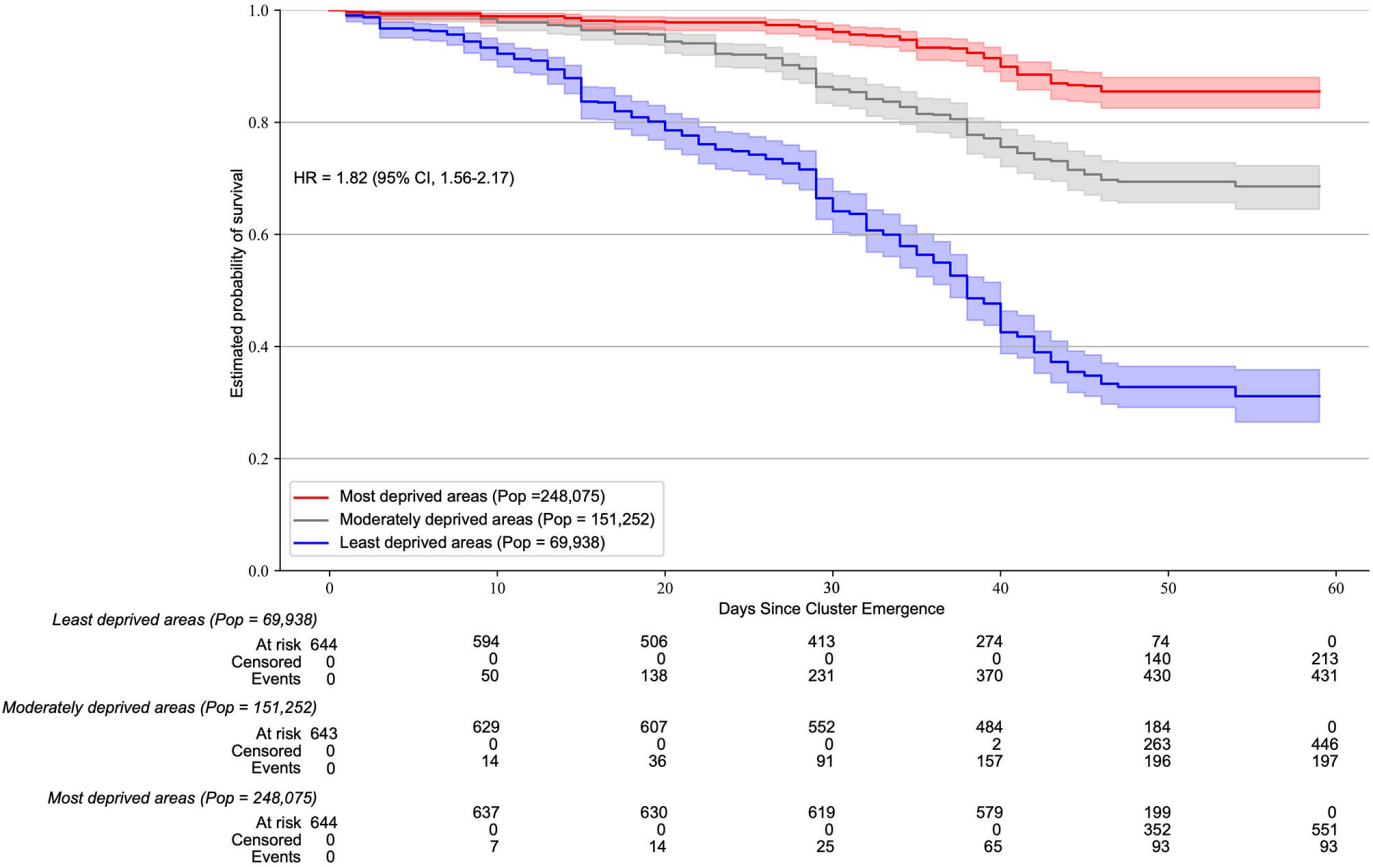
Neighborhood-level socioeconomic deprivation association with SARS-CoV-2 cluster persistence. Kaplan Meier survival estimates of SARS-CoV-2 clusters (600 m spatial window) according to terciles of the socioeconomic deprivation index. The hazard ratio and 95% CI correspond to a deprivation tercile change. The MST-DBSCAN algorithm was run with a maximum spatial distance of 600 m, a minimum time-distance value of 1 day, and a maximum time distance value of 14 days. Similar results were observed using different spatial windows (200, 400, 800, and 1,000 m).

We identified 1,079 spatial clusters over the 65 days study period, which, once projected covered, 1,931 neighborhoods of the state of Geneva (Figure 1). The median neighborhood-level SARS-CoV-2 incidence rate ranged from 0 cases per 100,000 (interquartile range, IQR = 650) in the least deprived tercile to 465 (IQR = 866) in the most deprived tercile. Clusters emerged on average 4 days earlier in the most deprived tercile compared to the moderately deprived and 6 days compared to the least deprived terciles (Supplementary Table 1). The persistence of clusters varied substantially across terciles of the neighborhood-level deprivation index (Figure 2). Two months after the emergence of SARS-CoV-2 clusters, almost 85% of the spatial clusters remained in the most deprived areas, compared to around 70% in the moderately deprived areas and around 30% in the least deprived areas. This trend was confirmed by the Cox PH model adjusted for neighborhood-level population density in which the standardized deprivation index was associated with an increased spatial cluster persistence (hazard ratio, HR = 1.43 [95% confidence interval, CI = 1.28–1.59], *P* < 0.005) and the adjusted tercile-specific deprivation index HR was 1.82 [95% CI, 1.56–2.17]. Hazard ratios and confidence intervals of the Cox PH model including the individual components of the socioeconomic deprivation index are presented in Table 1. Low median income, low median rent, high percentage of foreigners and high unemployment were associated with spatial cluster persistence to varying, but statistically significant, degrees (Table 1). There was no statistically significant association between spatial cluster persistence and tertiary education and the percentage of actives in the primary sector (Table 1).

## Discussion

We combined spatiotemporal cluster detection with survival analysis and found that neighborhood-level socioeconomic deprivation was associated with persistent spatial clustering of SARS-CoV-2. This result supports our hypothesis, suggesting that the increased risk of infection of disadvantaged communities may also be due to the persistence of community transmission. This suggestion is of importance, considering that socioeconomically disadvantaged individuals are also at risk of worse COVID-19 outcomes due to a greater burden of obesity and other chronic diseases (9). Moreover, digital COVID-19 public health tools, such as contact tracing apps, have been developed and deployed since the first phase of the pandemic. While data remain scarce, evidence suggests that socioeconomic status is a determinant of attitudes toward these technologies (10). Public health attention and locally tailored interventions are required in socioeconomically disadvantaged communities to prevent the intersectionality of these multiple aspects of disadvantage further compounding the risk of infection, the risk of serious illness and thus, of mortality (3, 11, 12).

### Limitations

The place of infection being unknown, we were not able to differentiate between persistence of spatial clusters driven by an increased local transmission or by an increased importation of cases in the community (e.g., from the workplace) or by a coalescence of both. We cannot exclude that the contact tracing strategy—which consisted in tracing and testing close contacts of positive cases—and socioeconomic variations in testing rate influenced spatial cluster persistence.

## Conclusions

These findings bring unique insights into the determinants of transmission and suggest that the increased risk of infection of disadvantaged individuals may also be due to the persistence of community transmission. The persistence of transmission in disadvantaged populations further highlights the pressing need for public health interventions preventing an exacerbation of inequalities in the risk of SARS-CoV-2 infection, of serious illness and thus, of mortality.

## Data Availability Statement

The dataset analyzed during the current study is available from the corresponding author upon reasonable request. The dataset could not be made publicly available due to the sensitivity of individual georeferenced SARS-CoV-2 testing data. Requests to access these datasets should be directed to Idris Guessous, idris.guessous@hcuge.ch.

## Author Contributions

DD performed the data analyses and drafted the manuscript. DD, IG, and SJ conceived the study and completed the manuscript. JS, NV, AA, and SS participated in the design of the study and helped to draft the manuscript. All authors read and approved the final manuscript.

## Conflict of Interest

The authors declare that the research was conducted in the absence of any commercial or financial relationships that could be construed as a potential conflict of interest.
