# Supplementary material for: Socioeconomically Disadvantaged Neighborhoods Face Increased Persistence of SARS-CoV-2 Clusters
Source: Front Public Health. 2021 Jan 27;8:626090. doi: 10.3389/fpubh.2020.626090 (PMC7894360; doi:10.3389/fpubh.2020.626090)
Supplement: Supplementary file 1 [file Table_1.docx]

**Table S1: Emergence of SARS-CoV-2 clusters (days since first case) according to terciles of the neighborhood-level deprivation index.** Statistical significance assessed with one-way ANOVA and Tukey’s (HSD) test.

| **Deprivation index - Terciles** | **Mean (SD)** |
| --- | --- |
| **Least deprived areas** | 21.8 (6.8) *** |
| **Moderately deprived areas** | 17.6 (6.1) *** |
| **Most deprived areas** | 15.8 (4.9) *** |

**** p < 0.001*
